# Supplementary material for: The Dual Prey-Inactivation Strategy of Spiders—In-Depth Venomic Analysis of Cupiennius salei
Source: Toxins (Basel). 2019 Mar 19;11(3):167. doi: 10.3390/toxins11030167 (PMC6468893; doi:10.3390/toxins11030167)
Supplement: Supplementary file 1 [file toxins-11-00167-s001.zip › Supplementary Fig S1.1-S1.19/Supplementary Fig S1.2.pdf]

Supplementary Fig S1.2

CLUSTAL O(1.2.4) multiple sequence alignment: Protein disulfide-isomerases

```
PDI_1_CUPSA                 MYIKLFLLS---LLALSIADEIKKENNVLVLTKDNFEEIVK-NKNVLVEFYAPWCGHCKA
PDI_2_CUPSA                 MYIKLFLLS---LLALSIADEIKKENNVLVLTKDNFEEIVK-NKNVLVEFYAPWCGHCKA
PDI_ALOMA                   MYIKLFVLS---LLAVSIADEVKRENNVLVLTKDNFEDTVK-NKNVLVEFYAPWCGHCKA
PDI_VIRFA                   MYIKLFLVS---LLSLSIADEIKRESNVLVLTKDNFEEAVK-NKNVLVEFYAPWCGHCKA
tr_T1L2F7_TETUR             -MIFHYSLSIALLVTLVSSADVKKEENVLVLNKSNFDGVVSENKYVLVEFYAPWCGHCKA
gi_xp_013790737_LYMPO       MWFRTFGLLFLCVVIAAGEDDIKKEENVLVLNKDNFEKVLKDNKYILVEFYAPWCGHCKA
                              :  : :    ::      ::*:*.*****.*.**:  :. ** :**************

PDI_1_CUPSA                 LEPEYAKAATILNEEKSELLLAKVDATAETELAEQHGVRGYPTIKFFREGKVLEYNGGRT
PDI_2_CUPSA                 LEPEYAKAATILNEEKSELLLAKVDATAETELAEQHGVRGYPTIKFFREGKVLEYNGGRT
PDI_ALOMA                   LEPEYAKAATTLNEEKSELLLAKVDATAETELAEQHGVRGYPTIKFFKEGKVLEYNGGRT
PDI_VIRFA                   LEPEYAKAATTLNEEKSELLLAKVDATAETELAEQHGVKGYPTIKFFKEGKVLEYNGGRT
tr_T1L2F7_TETUR             LAPEYAKAATSLANEGSEILLAKVDATEETDLAEKYEVRGYPTLKFFRNGKPTEYNGGRT
gi_xp_013790737_LYMPO       LAPEYAKAATQLADEKSDIKLAKVDATEETELAEKHGVRGYPTLKFFRDSEPVDYSGGRS
                            * ******** * :* *:: ******* **:***:: *:****:***::.:  :*.***:

PDI_1_CUPSA                 ADDIIRWLKKKTGPPANDLTTAEAAKTFVDSGEVVVVGFFKDQESPEAKIFKNVAAEMDD
PDI_2_CUPSA                 ADDIIRWLKKKTGPPANDLTTAEAAKTFVDSGEVVVVGFFKDQESPEAKIFKNVAAEMDD
PDI_ALOMA                   ADDIIRWLKKKTGPPANDLKTVDAAKSFVDSGEVVVVGFFKDQEGAEAKIFKNVAAEMDD
PDI_VIRFA                   ADDIIRWLKKKTGPPANDLSTVEAAKSLIDSGEVVVVGLFKNQDGPEAKIFKNVAAEMDD
tr_T1L2F7_TETUR             ADDLLKWVKKKTGPPAVTLESVEDANKLKESAEVAIIGFFKDQESDAAKAFLEVATQMDD
gi_xp_013790737_LYMPO       ADEIILWLKKKTGPPALTLKTVEETKQFKEGSEVVIIGFYKDLESKEAKIFLEAAAVTDD
                            **::: *:********  * :.: :: : :..**.::*::*: :.  ** * :.*:  **

PDI_1_CUPSA                 FVFGITSESSVYSDLKASKDGVILFKKFDEGRNEYEGELSEDDLKKFLKSNSLPVVVEFS
PDI_2_CUPSA                 FVFGITSESSVYSDLKASKDGVILFKKFDEGRNEYEGELSEDDLKKFLKSNSLPVVVEFS
PDI_ALOMA                   FVFGITSEDAVYSELKASKDGVILFKKFDEGRNEYEGELSEDDLKKFLKSNSLPVVVEFS
PDI_VIRFA                   FIFGITSDDAVYSELKASKDGVILFKKFDEGRNEYEGELTEDDVKKFLKSNSLPLVVEFS
tr_T1L2F7_TETUR             HPFGITNSEAVFGEFSVSKDGVVLFKKFDEKRNDLFEDITQENLKKFISINSLPLVVDFS
gi_xp_013790737_LYMPO       YPFAITSQDEIYKELDVQKDGIVLFKKFDEGKNVYEGDLTVDNVKKFVKANSLPLVVEFN
                            . *.**... :: ::...***::******* :*    ::: :::***:. ****:**:*.

PDI_1_CUPSA                 HETAQKIFGGDIKSHNLLFISKGSPDYESKIDVFRSVAKEFKSKVLFVTINTDDEDHERI
PDI_2_CUPSA                 HETAQKIFGGDIKSHNLLFISKGSPDYESKIDVFRSVAKEFKSKVLFVTINTDDEDHERI
PDI_ALOMA                   HETAQKIFGGDIKSHNLLFISKGSPDYESKIEIFRSVAKEFKSKVLFVTINTDDEDHERI
PDI_VIRFA                   HETAQKIFGGDIKAHNLLFISKGSSDYESKIEDFRTVAKEFKSKVLFVTINTDDEDHERI
tr_T1L2F7_TETUR             HETAQKIFGGEIKFHNLLFLSKKASSYEKLVEAFRKVAKEFRNKVLFVTINTDIEDHERI
gi_xp_013790737_LYMPO       HESAQKIFGGEIKSHNLLFISKTASDYEKILEDFRSVAKEFKSKVLFVTIDTDEEDHERI
                            **:*******:** *****:** : .**. :: **.*****:.*******:** ******

PDI_1_CUPSA                 MEFFGLKKDDAPTMRIIKLEEEMTKFKPLTTGITAEDIKGFVDGVLEGKIKQHLLSEDIP
PDI_2_CUPSA                 MEFFGLKKDDAPTMRIIKLEEEMTKFKPLTTGITAEDIKGFVDGVLEGKIKQHLLSEDIP
PDI_ALOMA                   MEFFGLKKEDAPTMRIIKLEEEMTKFKPLTTGITADEIKGFVTGVLEGKIKQHLLSEDIP
PDI_VIRFA                   MEFFGLKKEEAPSMRLIKLEEEMTKFKPLTTGITADDIRGFVSGVLEGKIKQHLLSEDVP
tr_T1L2F7_TETUR             MDFFGLKIEDAPGMRLIRLEEDMIKYKPETSDITEESILSFVQGVIDGKIKKHLLTQDVP
gi_xp_013790737_LYMPO       MEFFGLKKEDTPDMRLIVLEEEMNKFKPETTKITVENIRNFVQGVLDGKIKQHLLSQDIP
                            *:***** :::* **:* ***:* *:** *: ** :.* .** **::****:***::*:*

PDI_1_CUPSA                 EGWDKEPVKVLVGKNFDEVAFDKSKNVLVEFYAPWCGHCKQLAPIYDELGEKYKDNANVV
PDI_2_CUPSA                 EGWDKEPVKVLVGKNFDEVAFDKSKNVLVEFYAPWCGHCKQLAPIYDELGEKYKDNANVV
PDI_ALOMA                   EGWDKEPVKVLVGKNFDEVAFDKSKNVLVEFYAPWCGHCKQLAPIYDELGEKYKDNSNVV
PDI_VIRFA                   EGWDKESVKVLVGKNFDEVAFDKSKNVLVEFYAPWCGHCKQLAPIYDELGEKYKDNANVV
tr_T1L2F7_TETUR             SDWDKNPVKVLVGKNFDEVALDKSKDVLVEFYAPWCGHCKQLVPIYDALGEKYKDNSNVV
gi_xp_013790737_LYMPO       EDWDKNPVKVLVAKNFDEVAFDKSKDVLVEFYAPWCGHCKQLSPIYDELGAKYKDNDKVV
                            ..***: *****.*******:****:************** **** ** ***** :**

PDI_1_CUPSA                 IAKMDATGNELEHTKINSFPTIKLYKKDTNEVVEYNGERTLEGISQFIETDGEYGKAAPD
PDI_2_CUPSA                 IAKMDATGNELEHTKINSFPTIKLYKKDTNEVVDFNGERTLEGISRFIESGGVDGAAVKE
PDI_ALOMA                   IAKMDATANELEHTKINSFPTIKLYKKDTNEVVEYNGERTLEGISQFIETDGEYGKAAPD
```

```
PDI_VIRFA                 IAKMDATANELEHTKINSFPTIKLYKKDTNEVVDFNGERTLEGISRFIESGGVDGAAVKE
tr_T1L2F7_TETUR           IAKMDATANELEHTKINSFPTIKLFKKDTNEVLDFNGERTLEGLSQFIDSGGKLGASPKK
gi_xp_013790737_LYMPO     IAKMDATVNELEHTKINSFPTIKLYKRDTNEVVDFSGERTLDGLKNFIESGGVDGASPKE
                          ******* ****************:*:*****:::.*****:*:..**::.*  * :  .

PDI_1_CUPSA               EEEDEEE--EKDDEKAKRDEL
PDI_2_CUPSA               EEEDEEE--EKDDEKAKRDEL
PDI_ALOMA                 EEEDEEE--EKDDEKAKRDEL
PDI_VIRFA                 EEEDEEE--EKDDEKAKRDEL
tr_T1L2F7_TETUR           EEVDEEEEKEGKAD-PRKDEL
gi_xp_013790737_LYMPO     QEEEEEEEEGEDDDQKKRDEL
                          :* :***    . :  ::***
```

Percent Identity Matrix - created by Clustal2.1

1: PDI_1_CUPSA	68.15	72.12	91.92	92.73	100.00	97.78
2: PDI_2_CUPSA	67.14	70.30	89.70	94.95	97.78	100.00
3: PDI_AlOMA	67.55	70.30	90.71	100.00	92.73	94.95
4: PDI_VIRFA	68.15	71.52	100.00	90.71	91.92	89.70
5: tr_T1L2F7_TETUR	100.00	68.94	68.15	67.55	68.15	67.14
6: gi_xp_013790737_LYMPO	68.94	100.00	71.52	70.30	72.12	70.30

Proteomic coverage trypsin

>PDI_1_S1_CUPSA protein disulfide-isomerase isoform 1

MYIKLFLLSL LALSIA**DEIK KENNVLVLTK DNFEEIVKNK NVLVEFYAPW CGHCKALEPE YAKAATILNE EKSELLLAKV**
DATAETELAE QHGVRGYPTI KFFREGKVLE YNGGRTADDI IRWLK**KKTGP PANDLTTAEA AKTFVDSGEV VVVGFFKDQE**
SPEAKIFKNV AAEMDDFVFG ITSESSVYSD LKASKDGVIL FKKFDEGR**NE YEGELSEDDL KKFLKSNSLP VVVEFSHETA**
QKIFGGDIK**S HNLLFISKGS PDYESKIDVF R**SVAKEFK**SK VLFVTINTDD EDHERIMEFF GLKKDDAPTM RIIKLEEEMT**
KFKPLTTGIT AEDIKGFVDG VLEGKIKQHL LSEDIPEGWD KEPVKVLVGK NFDEVAFDKS KNVLVEFYAP WCGHCKQLAP
IYDELGEKYK DNANVVIAKM DATGNELEHT KINSFPTIKL YK**KDTNEVVE YNGERTLEGI SQFIETDGEY GK**AAPDEEED
EEEEKDDEKA KRDEL

>PDI_2_CUPSA protein disulfide-isomerase isoform 2

MYIKLFLLSL LALSIA**DEIK KENNVLVLTK DNFEEIVKNK NVLVEFYAPW CGHCKALEPE YAKAATILNE EKSELLLAKV**
DATAETELAE QHGVRGYPTI KFFREGKVLE YNGGRTADDI IRWLK**KKTGP PANDLTTAEA AKTFVDSGEV VVVGFFKDQE**
SPEAKIFKNV AAEMDDFVFG ITSESSVYSD LKASKDGVIL FKKFDEGR**NE YEGELSEDDL KKFLKSNSLP VVVEFSHETA**
QKIFGGDIK**S HNLLFISKGS PDYESKIDVF R**SVAKEFK**SK VLFVTINTDD EDHERIMEFF GLKKDDAPTM RIIKLEEEMT**
KFKPLTTGIT AEDIKGFVDG VLEGKIKQHL LSEDIPEGWD KEPVKVLVGK NFDEVAFDKS KNVLVEFYAP WCGHCKQLAP
IYDELGEKYK DNANVVIAKM DATGNELEHT KINSFPTIKL YKKDTNEVVD FNGERTLEGI SRFIESGGVD GAAVK**EEEED**
EEEEKDDEKA KRDEL

Proteomic coverage chymotrypsin

>PDI_1_S1_CUPSA protein disulfide-isomerase isoform 1

MYIKLFLLSL LALSIADEIK KENNVL**VLTK DNFEEIVKN**K NVLVEFYAPW **CGHCKALEPE YAKAA**TILNE EKSELL**LAKV**
DATAETELAE QHGVRGYPTI KFFREGKVLE YNGGRTADDI IRWLKKKTGP PANDLTTAEA AKTFVDSGEV VVVGFFKDQE
SPEAKIFKNV AAEMDDFVFG ITSESSVYSD LKASKDGVIL FKKFDEGRNE YEGEL**SEDDL KKFLKSNSLP VVVEFSHETA**
QKIFGGDIKS HNLLFISKGS PDYESKIDVF RSVAKEF**KSK VLFVTINTDD EDHE**RIMEFF GLKKDDAPTM R**IIKLEEEMT**
KFKPLTTGIT AEDIKGFVDG VLEGKIKQHL LSEDIPEGWD KEPVKVLVGK NFDEVAFDKS KNVLVEFYAP WCGHCKQLAP
IYDELGEKY**K DNANVVIAKM DATGNELEHT KINSFPTIKL YKKDTNEVVE YNGERTLEGI SQFIETDGEY GKAAPDEEED**
EEEEKDDEKA KRDEL

>PDI_2_CUPSA protein disulfide-isomerase isoform 2

MYIKLFLLSL LALSIADEIK KENNVL**VLTK DNFEEIVKN**K NVLVEFYAPW **CGHCKALEPE YAKAA**TILNE EKSELL**LAKV DATAETELAE QHGVRGYPTI KFFREGKVLE YNGGRTADDI IRWLKKKTGP PANDLTTAEA AKTFVDSGEV VVVGFFKDQE SPEAKIFKNV AAEMDDFVFG ITSESSVY**SD LKASKDGVIL FKKFDEGRNE YEGEL**SEDDL KKFLKSNSLP VVVEFSHETA QKIFGGDIKS HNLLFISKGS PDYESKIDVF** RSVAKEF**KSK VLFVTINTDD EDHE**RIMEFF GLKKDDAPTM R**IIKLEEEMT KF**KPLTTGIT AEDIKGFVDG VLEGKIKQHL LSEDIPEGWD KEPVKVLVGK NFDEVAFDKS KNVLVEFYAP WCGHCKQLAP IYDELGEKY**K DNANVVIAKM DATGNELEHT KINSFPTIKL YKKDTNEVVD FNGERTLEGI SRF**IE**SGGVD** G**A**A**VKE**EEED EEEEKDDEKA KRDEL

Supplementary Fig S1.2 Multiple sequence alignment of PDI from the venom glands of *C. salei* (PDI_1_CUPSA [MH766617, MH766618], PDI_2_CUPSA [MH766619]), *A. marikovskyi* (PDI_ALOMA), *V. fasciatus* (PDI_VIRFA), the horseshoe crab *L. polyphemus* (gi_XP_013790737_LYMPO), and the mite *Tetranychus urticae* (tr_ T1L2F7_TETUR). Signal peptides are marked in black characters, cysteines in white with a black box, positively charged amino acids in pink, negatively charged amino acids in blue, hydrophobic amino acids in red and polar amino acids in green. The disulfide isomerase domains (IPR005788) are highlighted in yellow and the thioredoxin conserved sites in gray.
